# Association of metabolic syndrome with the incidence of low-grade albuminuria: a cohort study in middle-aged and elderly Chinese adults

**DOI:** 10.18632/aging.202592

**Published:** 2021-03-03

**Authors:** Meng Ren, Lili You, Diaozhu Lin, Qiling Feng, Chulin Huang, Feng Li, Yiqin Qi, Wanting Feng, Chuan Yang, Li Yan, Kan Sun

**Affiliations:** 1 Department of Endocrinology, Sun Yat-sen Memorial Hospital, Sun Yat-sen University, Guangzhou 510120, People' s Republic of China

**Keywords:** metabolic syndrome, low-grade albuminuria, microalbuminuria, chronic kidney disease

## Abstract

Background: Individuals with metabolic syndrome have elevated risks of micro- and macro-albuminuria as well as chronic kidney disease (CKD).

Objective: To assess the influence of metabolic abnormalities on the presence of low-grade albuminuria (below the threshold for microalbuminuria).

Design, participants, and main outcome measures: This community-based cohort study included 3,935 eligible individuals aged 40 years or older. The presence of low-grade albuminuria was detected in those without micro- or macro-albuminuria and analyzed according to the highest quartile of the baseline urinary albumin-to-creatinine ratio (ACR ≥11.13 mg/g). CKD was defined by an estimated glomerular filtration rate <60 mL/min/1.73 m^2^ or the new presence of albuminuria (ACR ≥30 mg/g).

Results: Overall, 577 (14.7%) participants developed low-grade albuminuria and 164 (4.2%) participants developed CKD during a mean follow-up period of 3.6 years. Compared with participants without metabolic syndrome, those with metabolic syndrome had greater risks of low-grade albuminuria [adjusted odd ratio (OR) and 95% confidence interval (95% CI): 1.30 (1.05–1.61)] and CKD [1.71 (1.20–2.44)]. Moreover, the incidence rates of low-grade albuminuria and CKD increased as the number of metabolic syndrome components increased (P for trend <0.0001).

Conclusions: The presence of metabolic syndrome is associated with increased incidence rates of low-grade albuminuria and CKD the middle-aged and elderly Chinese populations.

## INTRODUCTION

Increasing evidence indicates that metabolic syndrome is associated with increased risks for development of albuminuria and chronic kidney disease (CKD) [1, 2]. The Third National Health and Nutrition Examination Survey (NHANES III) found that metabolic syndrome is a significant risk factor for the development of microalbuminuria and renal dysfunction, and some specialists have suggested that microalbuminuria can even be applied as a component of metabolic syndrome [3].

Albuminuria is now characterized as increased urinary albumin excretion, which is defined according to a spot urine albumin-to-creatinine ratio (ACR) ≥30 mg/g [4]. As an etiological factor for endothelial dysfunction, increased urinary albumin excretion is associated with many adverse cardiovascular events. However, multiple studies have indicated that a slight increase in albuminuria (i.e.,, low-grade albuminuria), which was previously considered to be urinary albumin excretion in the normal range (ACR <30 mg/g), could lead to an increased risk of cardiovascular disease and stroke [5, 6].

Even low levels of urinary albumin excretion can signify extensive vascular dysfunction and endothelial damage, which could be related to complications occurring in the context of metabolic abnormalities and cardiovascular diseases. However, the relationship between metabolic syndrome and mild albuminuria has not been thoroughly explored. A cross-sectional study in 9,579 middle-aged and elderly Chinese individuals found that low-grade albuminuria was significantly related with an increased prevalence of metabolic syndrome and its components [7]. In addition, another study in 202 non-diabetic African men reported that low-grade albuminuria is positively correlated with hypertension, one of the established components of metabolic syndrome [8]. Notably, these two studies had either a cross-sectional design or included a small sample. To our knowledge, no studies exploring the clinical impact of metabolic syndrome on the presence of mild albuminuria based on longitudinal data have been reported.

We hypothesized that metabolic abnormalities contribute significantly to the risk of low-grade albuminuria and progression of albuminuria. To test this hypothesis, we evaluated the associations of metabolic syndrome and the related components with the incidence rates of both low-grade albuminuria and CKD in a cohort of middle-aged and elderly Chinese individuals.

## RESULTS

### Clinical characteristics of the study population

Among the 3,935 eligible participants, the mean age of the cohort was 59.1±7.1 years. During a follow-up period of 3.6±0.7 years, 577 (14.7%) participants developed low-grade albuminuria and 164 (4.2%) developed CKD. With participants categorized by metabolic syndrome status at baseline, their clinical and biochemical characteristics at baseline are reported in Table 1. Compared with participants without metabolic syndrome, those with metabolic syndrome were older and had higher values for urinary ACR, eGFR, BMI, WC, SBP, DBP, TG, FPG, fasting insulin, γ-GGT, and HOMA-IR as well as a lower HDL-C level (all P<0.001). Low-grade albuminuria and CKD were present in 18.4% and 6.7% of those with metabolic syndrome, compared with 14.0% and 3.0% of those without metabolic syndrome, respectively (P<0.0005 for low-grade albuminuria and P<0.0001 for CKD).

**Table 1:** Baseline characteristics of study population according to metabolic syndrome status at baseline.

	**Without metabolic syndrome**	**With metabolic syndrome**	**P**
Metabolic syndrome [n (%)]	2691 (68.4)	1244 (31.6)	
Age (years)	54.9 ± 7.0	56.8 ± 7.3	< 0.0001
eGFR (ml/min per 1.73 m^2^)	103.0 ± 21.7	100.2 ± 20.5	0.0001
Urinary ACR (mg/g)	6.64 (5.01 – 8.33)	6.95 (5.29 – 8.61)	0.0002
Male [n (%)]	767 (28.5)	417 (33.5)	0.0014
BMI (kg/m^2^)	22.7 ± 2.9	25.1 ± 2.8	< 0.0001
WC (cm)	78.3 ± 8.4	86.8 ± 7.8	< 0.0001
SBP (mmHg)	120.2 ± 13.6	132.6 ± 15.3	< 0.0001
DBP (mmHg)	72.6 ± 8.9	78.8 ± 9.3	< 0.0001
Current smoking [n (%)]	243 (9.2)	123 (10.0)	0.4094
Current drinking [n (%)]	79 (3.0)	47 (3.9)	0.1619
TG (mmol/L)	1.08 (0.84 – 1.43)	1.86 (1.28 – 2.60)	< 0.0001
TC (mmol/L)	5.21 ± 1.22	5.11 ± 1.25	0.0119
HDL-C (mmol/L)	1.43 ± 0.35	1.12 ± 0.29	< 0.0001
LDL-C (mmol/L)	3.18 ± 0.93	3.06 ± 0.94	0.0004
FPG (mmol/L)	5.23 (4.91 – 5.56)	5.88 (5.44 – 6.37)	< 0.0001
Fasting insulin (μIU/ml)	6.20 (4.70 – 8.10)	9.10 (6.90 – 12.20)	< 0.0001
γ-GGT (U/L)	18.0 (14.0 – 25.0)	23.0 (17.0 – 33.0)	< 0.0001
Physical activity (MET-h/week)	24.0 (10.5 – 49.0)	22.8 (10.5 – 42.7)	0.1903
HOMA-IR	1.45 (1.09 – 1.96)	2.39 (1.79 – 3.34)	< 0.0001
Incident CKD [n (%)]	81 (3.0)	83 (6.7)	< 0.0001
Incident low-grade albuminuria [n (%)]	364 (14.0)	213 (18.4)	0.0005

Data were means ± SD or medians (interquartile ranges) for skewed variables or numbers (proportions) for categorical variables.

P values were for the ANOVA or χ^2^ analyses across the groups.

CKD, chronic kidney disease; ACR, albumin-to-creatinine ratio; BMI, body mass index; WC, waist circumference; SBP, systolic blood pressure; DBP, diastolic blood pressure; TG, triglycerides; TC, total cholesterol; HDL-C, high-density lipoprotein cholesterol; LDL-C, low-density lipoprotein cholesterol; FPG, fasting plasma glucose; eGFR, estimated glomerular filtration rate; γ-GGT, γ-glutamyltransferase; MET-h/week: separate metabolic equivalent hours per week; HOMA-IR, homeostasis model assessment of insulin resistance.

### Associations of metabolic syndrome with low-grade albuminuria and CKD

The adjusted ORs and 95% CIs for the risks of low-grade albuminuria and CKD according to presence of metabolic syndrome are presented in Table 2. Compared with the absence of metabolic syndrome, metabolic syndrome was independently associated with greater incidences of low-grade albuminuria (OR 1.39, 95% CI, 1.15–1.67) and CKD (OR 2.30, 95% CI, 1.68–3.15) on unadjusted logistic regression analyses. These associations of metabolic syndrome with low-grade albuminuria and CKD were attenuated, but persisted after adjustment for potential risk factors. After adjustment for age, sex, BMI, physical activity, smoking status, drinking status, γ-GGT, fasting insulin, eGFR, and ACR, the ORs and 95% Cis the associations of metabolic syndrome with low-grade albuminuria and CKD were 1.30 (1.05–1.61) and 1.71 (1.20–2.44), respectively. Examination of the internal consistency of the associations of metabolic abnormalities with incident low-grade albuminuria and CKD revealed that the incidences of low-grade albuminuria and CKD were significantly higher in participants with an increased number of metabolic syndrome components on both unadjusted and multivariate adjusted logistic regression analyses (all P for trend values <0.0001; Table 3).

**Table 2:** The risk of incident low-grade albuminuria and CKD according to metabolic syndrome status at baseline.

		**Without metabolic syndrome**	**With metabolic syndrome**	**P**
Low-grade albuminuria	Model 1	1	1.39 (1.15 – 1.67)	0.0005
	Model 2	1	1.36 (1.13 – 1.65)	0.0012
	Model 3	1	1.28 (1.04 – 1.57)	0.0182
	Model 4	1	1.30 (1.05 – 1.61)	0.0167
CKD	Model 1	1	2.30 (1.68 – 3.15)	< 0.0001
	Model 2	1	2.06 (1.50 – 2.83)	< 0.0001
	Model 3	1	1.78 (1.27 – 2.51)	0.0009
	Model 4	1	1.71 (1.20 – 2.44)	0.0030

Data are hazard ratio (95% confidence interval). Participants without low-grade albuminuria or CKD are defined as 0 and with low-grade albuminuria or CKD as 1.

Data are hazard ratio (95% confidence interval) compared with subjects without metabolic syndrome.

Model 1 is unadjusted.

Model 2 is adjusted for age and sex.

Model 3 is adjusted for age, sex, BMI, physical activity, smoking status, drinking status.

Model 4 is adjusted for age, sex, BMI, physical activity, smoking status, drinking status, γ-GGT, fasting insulin, eGFR and ACR.

**Table 3:** The risk of incident low-grade albuminuria and CKD according to different number of metabolic syndrome components.

		**Number of metabolic syndrome components**					**P for trend**
		**0 component**	**1 component**	**2 components**	**3 components**	**≥ 4 components**	
Low-grade albuminuria	Model 1	1	1.18 (0.85 – 1.63)	1.05 (0.76 – 1.46)	1.63 (1.19 – 2.24)	1.96 (1.41 – 2.73)	< 0.0001
	Model 2	1	1.15 (0.83 – 1.59)	1.03 (0.74 – 1.43)	1.59 (1.15 – 2.19)	1.85 (1.32 – 2.59)	< 0.0001
	Model 3	1	1.15 (0.82 – 1.62)	1.05 (0.74 – 1.49)	1.58 (1.11 – 2.23)	1.86 (1.28 – 2.69)	< 0.0001
	Model 4	1	1.17 (0.83 – 1.64)	1.09 (0.77 – 1.55)	1.66 (1.16 – 2.36)	2.00 (1.37 – 2.92)	< 0.0001
CKD	Model 1	1	1.97 (0.79 – 4.91)	2.95 (1.22 – 7.09)	6.16 (2.64 – 14.37)	6.91 (2.93 – 16.30)	< 0.0001
	Model 2	1	1.76 (0.71 – 4.41)	2.58 (1.07 – 6.22)	5.17 (2.21 – 12.10)	5.68 (2.39 – 13.47)	< 0.0001
	Model 3	1	1.90 (0.71 – 5.12)	2.55 (0.97 – 6.66)	4.77 (1.87 – 12.17)	4.98 (1.91 – 12.96)	< 0.0001
	Model 4	1	1.82 (0.68 – 4.92)	2.36 (0.90 – 6.20)	4.40 (1.71 – 11.30)	4.53 (1.72 – 11.91)	< 0.0001

Data are hazard ratio (95% confidence interval). Participants without low-grade albuminuria or CKD are defined as 0 and with low-grade albuminuria or CKD as 1.

Data are hazard ratio (95% confidence interval) compared with subjects with 0 component of metabolic syndrome.

Model 1 is unadjusted.

Model 2 is adjusted for age and sex.

Model 3 is adjusted for age, sex, BMI, physical activity, smoking status, drinking status.

Model 4 is adjusted for age, sex, BMI, physical activity, smoking status, drinking status, γ-GGT, fasting insulin, eGFR and ACR.

### Subgroup analyses for associations of metabolic syndrome with low-grade albuminuria and CKD

We conducted stratified analyses to determine the differential risks of metabolic syndrome and CKD in different subgroups. Multivariate analyses of the subgroups indicated that the association of metabolic syndrome with CKD was consistent, except in the subgroup of participants with diabetes. Additionally, the relationship of metabolic syndrome with CKD was inconsistent in the subgroup with diabetes (P for interaction = 0.0331), and a significantly higher incidence of CKD associated with metabolic syndrome was observed in subgroups without diabetes. As shown in Table 4.

**Table 4:** The risk of incident low-grade albuminuria and CKD according to metabolic syndrome status at baseline in different subgroups.

		**n, case/subjects**	**Without metabolic syndrome**	With metabolic syndrome^*^	**P for interaction**
Low-grade albuminuria	Age				0.1829
	≥ 60	250/1520	1	1.48 (1.08 – 2.02)	
	< 60	327/2251	1	1.16 (0.86 – 1.57)	
	Sex				0.6799
	Men	134/1119	1	1.48 (0.96 – 2.27)	
	Women	443/2652	1	1.24 (0.97 – 1.59)	
	BMI				0.1370
	Normal	326/2308	1	1.54 (1.14 – 2.09)	
	Overweight	202/1184	1	1.10 (0.79 – 1.54)	
	Obesity	49/279	1	1.01 (0.49 – 2.07)	
	Diabetes				0.7636
	Yes	151/758	1	1.36 (0.90 – 2.07)	
	No	426/3013	1	1.14 (0.88 – 1.49)	
	Hypertension				0.1419
	Yes	164/703	1	1.58 (1.06 – 2.35)	
	No	413/3068	1	1.00 (0.76 – 1.32)	
CKD	Age				0.6897
	≥ 60	98/1618	1	1.84 (1.14 – 2.96)	
	< 60	66/2317	1	1.47 (0.82 – 2.62)	
	Sex				0.7546
	Men	65/1184	1	2.02 (1.12 – 3.63)	
	Women	99/2751	1	1.47 (0.93 – 2.32)	
	BMI				0.6874
	Normal	71/2379	1	1.67 (0.97 – 2.87)	
	Overweight	70/1254	1	1.32 (0.77 – 2.28)	
	Obesity	23/302	1	4.92 (1.28 – 18.97)	
	Diabetes				0.0331
	Yes	52/810	1	1.12 (0.58 – 2.15)	
	No	112/3125	1	1.82 (1.17 – 2.83)	
	Hypertension				0.3328
	Yes	63/766	1	1.43 (0.77 – 2.67)	
	No	101/3169	1	1.56 (0.98 – 2.48)	

^*^Data are hazard ratio (95% confidence interval). Participants without low-grade albuminuria or CKD are defined as 0 and with low-grade albuminuria or CKD as 1. The model is adjusted for age, sex, BMI, physical activity, smoking status, drinking status, γ-GGT, fasting insulin, eGFR and ACR.

## DISCUSSION

In this study of 3,935 Chinese participants aged 40 years or older, the presence of metabolic syndrome significantly increased the participants’ risks of low-grade albuminuria and CKD. Monitoring and management of early renal damage should be emphasized among middle-aged to elderly Chinese individuals, especially those with more than one metabolic abnormality. To our knowledge, this was the first and largest cohort study to investigate the associations of metabolic syndrome with incident low-grade albuminuria.

We speculate that the increased rates of low-grade albuminuria and CKD in study participants with metabolic syndrome may be related to sustained deterioration in metabolic abnormalities such as insulin resistance, hypertension, obesity, and dyslipidemia. This hypothesis is supported by previous studies demonstrating that each single component of metabolic syndrome is associated with a significant increase in albuminuria risk [9–11]. Although low-grade albuminuria portends an increased risk of future cardiovascular diseases and related death, no previous long-term studies have determined whether a potential risk for low-grade albuminuria exists in those with metabolic syndrome. In this study, we found that that presence of metabolic syndrome did increase the risk of low-grade albuminuria, a finding that can provide valuable insight into the definition of normal reference values for urine albumin excretion. Moreover, increasing numbers of metabolic syndrome components were positively associated with increased risk of low-grade albuminuria. We therefore hypothesize that a series of metabolic abnormalities and their coexisting conditions is associated with a greater likelihood of kidney dysfunction and damage, even at a very early stage.

The association of metabolic syndrome with the risks of low-grade albuminuria and CKD can be explained by several mediating factors and biologic hypotheses. The presence of each metabolic syndrome component is independently associated with the progression of microangiopathy in the kidney. Mechanistically, elevated blood pressure, increased insulin resistance, and related abnormal glucose metabolism are directly associated with endothelial dysfunction and renal hemodynamic instability, leading to podocyte injury and consequently albuminuria in the glomerulus [11, 12]. Obesity is accompanied by glomerular hyperfiltration, which was shown to be an independent predictor of incident albuminuria [13]. Recent studies found that obesity and hypertension can impair renal afferent arteriolar autoregulation, reduce podocyte density, and synergistically contribute to the development of albuminuria [14, 15]. Moreover, abnormal lipid profiles can accelerate atherosclerosis through renovascular and fat deposition in renal tubules, which leads to endothelial cell inflammation and tubulointerstitial damage [16].

In addition, as shown by the results of our subgroup analyses, the increased risk of low-grade albuminuria associated with metabolic syndrome was significant in the normal group, while the increased risk of CKD was significant in the obesity group. First, no statistically significant interaction term was found between metabolic syndrome and BMI stratification for the development of low-grade albuminuria and CKD. Moreover, metabolic syndrome was positively related with the risks of low-grade albuminuria and CKD in all BMI subgroups in this study. Our previous study [17] demonstrated significant associations of BMI with low-grade albuminuria and CKD, and an overweight or obese status is closely related to inflammation and insulin resistance [18–20]. We assume that a high BMI and the related inflammation and insulin resistance aggravate the relationship between metabolic syndrome and CKD. However, the potential pathophysiological mechanisms linking low-grade albuminuria or CKD to obesity are not fully established. Obesity can increase the risk of CKD development via hormonal, hemodynamic, and metabolic changes that occur in the body with a high level of fat accumulation [21]. On the other hand, obesity is a major risk factor for hypertension and type 2 diabetes, which are closely related with a high prevalence of CKD [22]. Therefore, we suggest that further research exploring the associations of BMI, metabolic syndrome, and kidney injury is needed.

Some limitations of the study should be considered. First, although the definition of CKD remains unchanged, the presence of altered creatinine and urinary albumin excretion for more than 3 months is still part of the CKD definition. However, we defined both CKD and low-grade albuminuria on the basis of a single serum creatinine measurement and the urinary ACR level, which may have resulted in overestimation of the incidence of disease. Second, the follow-up rate is important for assessing the validity of a dataset for research purposes. The follow-up rate was 70.6% in this study, which is relatively low and may reduce the reliability of the study. Third, additional analyses considering more detailed data including previous nephropathy history, personal income level, antihypertensive drug use, and dietary protein consumption would strengthen the findings of the present study. Fourth, our findings are also limited by the lack of long-term follow-up, which could provide more information regarding a moderate number of outcome events.

In conclusion, our data indicate that the presence of metabolic syndrome increases the risk of low-grade albuminuria in Chinese adults older than 40 years. Moreover, our study raises individual and public health concerns regarding the influence of metabolic syndrome on kidney health. Additional prospective studies in other populations and ethnic groups are needed to support our findings.

## MATERIALS AND METHODS

### Study participants

**Figure 1:**
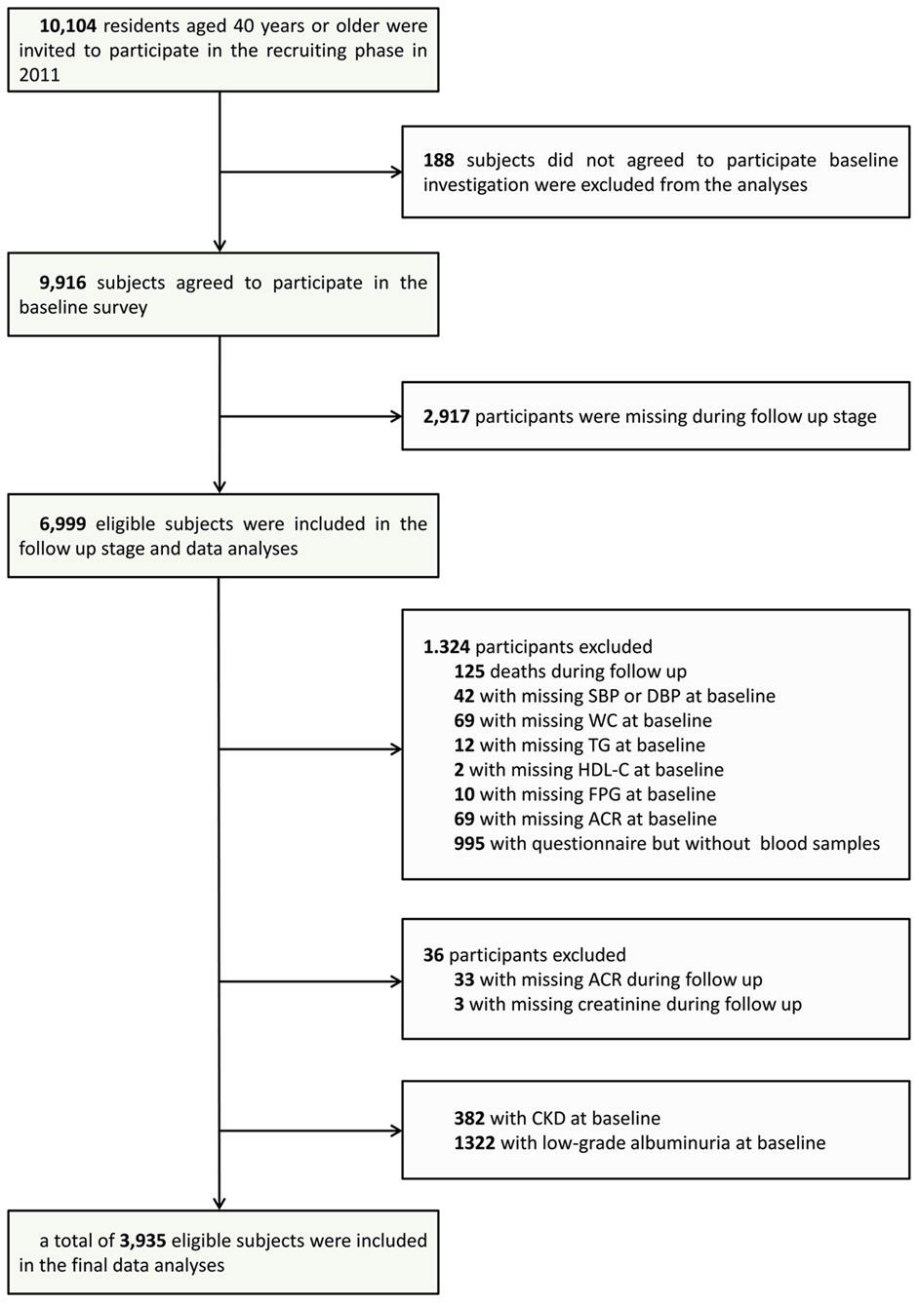
**Flowchart of the population selection of the study.**

The participants for this study were collected from the Risk Evaluation of Cancers in Chinese Diabetic Individuals: A Longitudinal Study (the REACTION Study), and the detailed characteristics of these individuals are available in the literature [23–25]. All participants were 40 years of age or older and had self-care ability. Participants in the present prospective cohort study were recruited from one center in Guangzhou, China from June to November, 2011 according to the process outlined in the study flow diagram (Figure 1). A total of 10,104 qualifying residents were invited to participate through examination notices or home visits, and 9,916 individuals provided written consent for participation in the baseline survey, for a participation rate of 98.1%. For the data analysis, 382 individuals with CKD and 1322 individuals with low-grade albuminuria at baseline were excluded. A total of 2,917 participants were lost to follow-up, and thus, the follow-up rate in this study was 70.6%. Participants who did not provide all required information (n=1,360) were also excluded from the analyses. Accordingly, a total of 3,935 eligible participants were included in the final data analyses.

The protocol for the present study was approved by the Institutional Review Board of Sun Yat-sen Memorial Hospital, Sun Yat-sen University and conformed to the principles of the Declaration of Helsinki II. All participants provided written informed consent prior to data collection.

### Questionnaire investigation

The data collected for each participant via a standardized questionnaire included lifestyle factors, family history, and sociodemographic characteristics. Smoking and alcohol consumption habits were classified as ‘never’, ‘current’ (smoking or drinking regularly within the previous 6 months), or ‘ever’ (the individual had stopped smoking or drinking more than 6 months before the study) [26]. Physical activity during leisure time was estimated using a short form of the International Physical Activity Questionnaire (IPAQ) with added questions related to the frequency and duration of moderate or vigorous activities and walking [27]. For the evaluation of overall physical activity, we calculated metabolic equivalent hours per week (MET-h/week) for each participant.

### Clinical and biochemical measurements

Anthropometrical measurements were collected for all participants by trained staff applying standard protocols. Body height and weight were recorded to the nearest 0.1 cm and 0.1 kg, respectively. Body mass index (BMI) was calculated by dividing weight by height squared (kg/m^2^). According to the standard for the local population, obesity was defined by a BMI of 28 kg/m^2^ or greater, and overweight was defined by a BMI equal to or exceeding 24 kg/m^2^ but less than 28 kg/m^2^ [28–31]. With participants in standing position, waist circumference (WC) was measured at the umbilical level after gentle expiration. Participants’ blood pressure was measured three times with a 5-minute interval used an automated blood pressure monitor (OMRON, Omron Company, China). The average values from the three measurements for systemic blood pressure (SBP) and diastolic blood pressure (DBP) were used in subsequent analysis. Venous blood samples were collected after overnight fasting for a minimum of 10 hours, and an autoanalyzer (Beckman CX-7 Biochemical Autoanalyzer, Brea, CA, USA) was used to measure the levels of fasting plasma glucose (FPG), fasting serum insulin, triglycerides (TG), high-density lipoprotein cholesterol (HDL-C), low-density lipoprotein cholesterol (LDL-C), total cholesterol (TC), creatinine, and γ-glutamyltransferase (γ-GGT) for each participant. As an index of insulin resistance, the homeostasis model assessment of insulin resistance (HOMA-IR) value was calculated as previously described [32].

### Definitions of low-grade albuminuria and CKD

Albuminuria was defined using the most recent guidelines of the American Diabetes Association’s Standards of Medical Care [33]. Albumin and creatinine concentrations were measured in first morning spot urine samples by chemiluminescence immunoassay (Siemens Immulite 2000, USA) and by the automatic analyzer using Jaffe’s kinetic method (Biobase-Crystal, Jinan, China), respectively. The urinary ACR was then calculated and expressed in units of mg/g. Albuminuria was defined by a urinary ACR of 30 mg/g or greater. In participants without albuminuria, low-grade albuminuria was defined according to the highest quartile of the baseline urinary ACR (≥11.13 mg/g).

The estimated glomerular filtration rate (eGFR) was calculated using the abbreviated Modification of Diet in Renal Disease (MDRD) formula recalibrated for the Chinese population: eGFR (ml/min per 1.73 m^2^) = 175 × [serum creatinine (μmol/L) × 0.011]^-1.234^ × [age]^-0.179^ × [0.79 if female] [34]. CKD was defined by an eGFR less than 60 mL/min per 1.73 m^2^ or new presence albuminuria (ACR ≥ 30 mg/g) [35].

### Definition of metabolic syndrome

In the present study, we applied a harmonized definition of metabolic syndrome provided in a joint statement from multiple relevant scientific organizations [36]. Specifically, participants were diagnosed with metabolic syndrome if at least three of the following abnormal conditions were observed: (1) serum TG ≥1.7 mmol/L indicating hypertriglyceridemia; (2) reduced HDL-C <1.0 mmol/L for men or <1.3 mmol/L for women; (3) increased blood pressure ≥130/85 mmHg; (4) elevated FPG ≥5.6 mmol/L or drug treatment for high blood glucose concentrations; and (5) WC ≥85 cm for men and ≥80 cm for women (recommended cutoff points for the Chinese population) [37].

### Statistical analysis

The data for continuous variables are presented as mean ± standard deviation (SD), and those for skewed variables are presented as median (interquartile range). Data for categorical variables are expressed as number (proportion). Differences in clinical characteristics and laboratory variables among groups were tested by one-way analysis of variance (ANOVA). Comparisons of categorical variables between groups were performed with the χ^2^ test. Linear regression analyses were performed to identify trends across groups.

Unadjusted and multivariate-adjusted logistic regression analyses were performed to evaluate the risk of low-grade albuminuria and CKD in relation to the presence of metabolic syndrome. The results of these analyses are presented as odd ratios (ORs) with 95% confidence intervals (95% CIs). Covariates in the fully adjusted logistic regression models were generated using a previously described method along with potential intermediate factors associated with albuminuria progression [38]. Model 1 included no adjustments, whereas Model 2 included adjustments for age and sex. Model 3 was adjusted for age, sex, BMI, physical activity, smoking status, and drinking status, and in addition to these factors, Model 4 was adjusted also for γ-GGT, fasting insulin, eGFR, and ACR.

We tested the direct associations of the number of metabolic syndrome components with the risks of low-grade albuminuria and CKD via logistic regression analysis. The number of metabolic syndrome components was classified as: 0 (reference), 1, 2, 3, and ≥4. In subgroup logistic regression analyses, the associations of metabolic syndrome with low-grade albuminuria and CKD were examined using a fully adjusted model with stratification of participants by age (≥60/<60 years), sex (male/female), obesity status during follow-up (normal/overweight/obesity), presence of diabetes during follow-up (yes/no), and presence of hypertension during follow-up (yes/ no). Interaction tests were performed by simultaneously including the interaction terms (strata variable multiplied by metabolic syndrome status), each strata factor, and metabolic syndrome status in the logistic regression analyses.

SAS version 9.3 software (SAS Institute Inc, Cary, NC, USA) was used for all statistical analyses, and all statistical tests were two-sided, with P<0.05 indicating a statistically significant difference.

### Statement of authorship

All authors believe that the manuscript represents valid work and have reviewed and approved the final version. The work has not been published previously and is not under consideration for publication elsewhere, in part or in whole.

### Compliance with ethical standards

The protocol for the present study involving human participants was approved by the Institutional Review Board of Sun Yat-sen Memorial Hospital, Sun Yat-sen University and followed the principles of the Declaration of Helsinki II. Prior to data collection, each patient provided written informed consent for the use of their data in this study.

### Data availability

The raw data are available upon request sent to the following E-mail: skendo@163.com.
